# Provision of training for accommodation providers in the London Borough of Hackney – results of a scoping exercise and educational session pilot

**DOI:** 10.1192/bjo.2021.356

**Published:** 2021-06-18

**Authors:** Kate Aldersey, Sheraz Ahmad, Sian Mason

**Affiliations:** East London NHS Foundation Trust

## Abstract

**Aims:**

To understand the level of training given to staff in providers of accommodation in the London Borough of Hackney across mental and physical health.

**Method:**

The Urgent and Emergency Care Collaborative (Health Education England) put out a call for funding bids around a number of workforce priority areas. This included upskilling care home staff to reduce admissions. We considered care home staff as those working across supported living schemes, housing with care, residential and nursing homes. Some of these settings exclusively support people with mental health needs.

We obtained a list of accommodation providers across the borough via the Local Authority. As a Community Rehabilitation team we work closely with many of the providers. We also co-facilitate the Hackney Mental Health Supported Accommodation panel and review all funded placements annually. We made contact via email and phonecall and arranged face to face meetings with 11 providers. We asked a standard set of questions about the organisation and training provision. We also asked them to identify gaps in training.

**Result:**

The level of training provided to staff varies vastly across different settings. There was a predominance of e-learning for some providers. Most staff in mental health settings are support worker level which limits the level of training offered/received.

Providers varied greatly in size of project and management structure and this directly impacts on access to training, often as a result of cost.

Providers were able to identify training gaps and were keen to have additional training.

Some common themes emerged – dual diagnosis, psychosis, medication – and some setting specific themes – dementia.

Based on the gaps identified we provided training sessions to a total of ~40 staff across a number of settings. Content included mental health awareness, crisis signposting and medicines management. All sessions were well received with pre and post-training questionnaires demonstrating an improvement in knowledge and confidence.

**Conclusion:**

There is potential for knowledge sharing across accommodation settings and for stronger links between accommodation providers and healthcare providers. We plan to explore the possibility of quantitative data on the number of Emergency Department presentations from accommodation settings locally.

